# Language and Thought in the Motion Domain: Methodological Considerations and New Empirical Evidence

**DOI:** 10.1007/s10936-019-09668-5

**Published:** 2019-09-03

**Authors:** Diego Feinmann

**Affiliations:** 1 Department of Philosophy, University of Sheffield, 45 Victoria Street, Sheffield, S3 7QB UK; 2 Department of Theoretical and Applied Linguistics, University of Cambridge, Cambridge, UK

**Keywords:** Linguistic relativity, Whorf, Motion cognition, Path, Manner, Similarity ratings, Verbal interference, English, Spanish

## Abstract

This study investigates whether there is a relation between how motion is linguistically expressed and how it is conceptualised. To do this, native speakers of two languages that differ typologically in how they encode telic motion (English and Spanish) are compared in both a verbal and a non-verbal experiment. The preferred non-verbal methods to test the linguistic relativity hypothesis in this domain have so far been recognition memory and binary judgments. This study questions the experimental validity of these approaches and implements an alternative method which combines similarity ratings with a verbal interference manipulation. The results reported here constitute evidence against linguistic relativity and in support of cognitive universalism.

## Introduction

Classical theories of cognition generally take humans’ conceptual structure to be universal, that is, invariant across languages and cultures. This position is represented by the work of, among others, Fodor (1975), Jackendoff (1983, 1990), Gleitman and Papafragou (2005) and Pinker (1994, 2007). Over the last three decades, a group of psychologists, linguists and anthropologists, chief among whom are Lucy (1992a, b, 1996, 2010), Levinson (1996, 1997, 2003) and colleagues (e.g. Gumperz and Levinson 1996; Bowerman and Levinson 2001), and Boroditsky (2001, 2011) and colleagues (Winawer et al. 2007; Casasanto and Boroditsky 2008; Fuhrman et al. 2011; Boroditsky et al. 2011; Thibodeau and Boroditsky 2013), have revived interest in an alternative view, known as the linguistic relativity (or Sapir-Whorf) hypothesis. Though by no means a monolithic group, these researchers share the belief that, to paraphrase Lucy (1992b: 3), ‘there may be some identifiable cognitive correlates (outside of the specifically linguistic realm) associated with using a particular language’. This position is supported by a significant number of studies which suggest that, at least in some domains, language-specific categories may indeed exert an influence on thought.

### Testing the Linguistic Relativity Hypothesis

Linguistic relativity research begins with the following question: to the extent that languages differ, is it the case that speakers of different languages have different thoughts about reality? This question is difficult to answer, and multiple factors may be responsible. First, ‘thought’ is not a singular cognitive phenomenon and comprises an array of informational processes; thus, in the same way as ‘reality’ or ‘the universe’, ‘thought’ is too vague a category and needs to be refined conceptually before being subjected to scientific scrutiny. Second, although languages differ in many significant ways, there is no obvious reason to decide a priori which of these differences may bear an influence on thought. More complexity is added by the fact that language and thought are rarely experienced as separate phenomena, which makes it difficult to treat them experimentally as different variables.

In spite of these difficulties, researchers have tried to shed light on this issue by investigating whether differences in non-verbal behaviour are causally related to language-specific differences. The rationale of this approach could be summarised as follows: assuming that behaviour is a reflection of a reasoning or thinking process, and if it is true that certain lexico-grammatical features can have an influence on thought, speakers of two different languages which happen to encode one domain of experience in a markedly different way from one another may also behave differently when tested in that particular domain in a non-verbal task.

Many researchers who adopted this modus operandi have claimed to find positive evidence for linguistic relativity. For example, in a series of studies concerned with grammatical influence on non-verbal behaviour, Lucy (1992a; Lucy and Gaskins 2001, 2003) has shown that speakers of non-plural marking classifier languages (e.g. Yucatec) are more likely to base their similarity judgements of objects on substance properties than speakers of languages with obligatory grammatical number marking and count-mass noun distinction (e.g. English), who tend to base their judgments on shape. In the domain of colour, Winawer et al. (2007) tested English and Russian speakers, and found that an obligatory colour distinction in Russian between *siniy* (‘dark blue’) and *goluboy* (‘light blue’) led to differences in colour discrimination, i.e.: Russian speakers were faster to discriminate two colours when they belonged to different linguistic categories in Russian (one to *siniy* and the other to *goluboy*) than when they fell under the same linguistic category. Spatial and object cognition has been another area of active research. Levinson (1994, 1996, 1997) and Haun et al. (2011), for instance, found that speakers whose respective languages differ in how they represent spatial location behave differently when asked to reproduce certain arrays of objects non-verbally; more recently, Koster and Cadierno (2018) found that German speakers have better recognition memory for object position than Spanish speakers.^1^

It is unclear, however, whether these studies can be taken as evidence for linguistic relativity. To explain why this is the case, it first needs to be made clear what counts (and what does not count) as evidence for this hypothesis. The core belief behind relativity is that the habitual use of language can have *enduring* effects on cognition; Levinson (2003: 291), for example, maintains that ‘[…] the need to output language coded in specific semantic parameters can force a deep-seated specialization of mind’. According to this view (see also Lucy 1992a, 1992b; Boroditsky 2001; Majid et al. 2004; Haun et al. 2011), the habitual use of language has the capacity to induce a given mode of mental processing, which may persist even in circumstances in which language is not being used. This is why evidence for linguistic relativity is often discussed in the literature in terms of ‘outside speech time’ (Cardini 2010) or ‘after language’ (Wolff and Holmes 2011) effects.

Given this, linguistic relativity researchers have been generally concerned, as Dan Slobin points out, ‘with the search for influences of particular languages on non-linguistic condition *in situations in which language is not being used, overtly or covertly*’ (Slobin 2003: 157, his emphasis). Though most of the data presented in support of linguistic relativity have been obtained in non-verbal tasks (e.g. similarity judgments and memory recognition), it is actually plausible that participants, when engaged in these experiments, could have made use of internal verbal descriptions of the target to solve the task given (for example, in the domain of colour, using colour names to guide choices). This evidence, therefore, should be approached with caution. Opponents of relativity, in fact, typically dismiss the results obtained in behavioural experiments, arguing that language is likely to have covertly mediated participants’ performance and, therefore, contaminated what was supposed to be a non-verbal task (e.g. Pinker 1994; Papafragou et al. 2002).

It should be noted that Slobin (1996a) has hypothesised about one type of linguistic effect on cognition known as ‘thinking for speaking’, which is distinct from and less controversial than linguistic relativity (cf. Levelt 1989; Pinker 1994); this effect, however, is often referred to in the literature as a ‘weak’ form of relativity (e.g. Han and Cadierno 2010; Cardini 2010; Filipović 2011). This can be confusing because, from a theoretical viewpoint, ‘thinking for speaking’ is not a Whorfian effect but rather a phenomenon believed to occur at the thought/language interface. Indeed, ‘thinking for speaking’ effects are hypothesised to arise under very specific conditions, that is, prior to the moment of speaking, when thoughts are believed to structure themselves according to the structure of the language to be spoken. Thus, ‘thinking for speaking’, for which there is some experimental evidence (e.g. von Stutterheim and Nüse 2003; Papafragou et al. 2008), is a transient phenomenon; that is, unlike linguistic relativity, it is not hypothesised to extend beyond speech time.

## The Conceptualisation of Motion Events

Over the past 15 years much linguistic relativity research has been devoted to studying the relationship between how motion events are expressed cross-linguistically and how they are conceptualised across speakers of different languages. This type of research draws mainly on Talmy’s (1985) typological work on motion events, which divided the languages of the world into two groups according to how motion events are lexicalised. In what follows, I will review the relevant typological work done on motion lexicalisation, as well as the attempts made to test the linguistic relativity hypothesis in this domain.

### The Typology of Motion Expressions: The Case of English Versus Spanish

Motion is a dynamic domain of experience which involves the movement of an entity through space. According to Talmy (2000: 25–27), ‘motion’ is composed of four basic semantic components: a *Figure* (or the moving entity), a *Ground* (or spatial reference), the fact of *Motion* and the *Path* or trajectory followed by the moving *Figure*. In addition to these basic components, information relating to the *Manner* of displacement (e.g. walking, running) is commonly lexicalised too.

Talmy (1985) proposed to divide the world languages into two groups on the basis of how languages encode the semantic components of the motion event. In the so-called ‘satellite-framed’ languages (or S-languages), Path information tends to be expressed in a ‘satellite’ or verb particle attached to the clause’s main verb whereas Manner information is encoded in the main verb. Typical S-languages include most Indo-European languages as well as Finno-Ugric (e.g. Finish, Hungarian) and Sino-Tibetan (e.g. Mandarin Chinese) languages (Talmy 1991). The following English sentence illustrates how S-languages typically encode a motion event:^2^

**Table:** 

(1) *Boris* [F]	*walked* [Mo + Ma]	*across* [P]	*Trafalgar Square* [G]

In ‘verb-framed’ languages (or V-languages), by contrast, Path information is usually expressed in the main verb of the clause whereas Manner information is either expressed at the end of that clause in the form of an adjunct complement or omitted altogether. V-languages include Romance (e.g. Spanish, Italian, French), Semitic (e.g. Hebrew, Moroccan Arabic) and Turkic languages, among others (Talmy 1991). The following Spanish sentence illustrates how V-languages typically encode a motion event:

**Table:** 

(2) *Boris* [F]	*cruzó* [Mo + P]	*Trafalgar Square* [G]	*caminando* [Mo + Ma] (optional)	(Spanish)
‘Boris	crossed	Trafalagar Square	walking’	

Because the expression of Manner information is optional in V-languages, speakers of V-languages like Spanish tend to indicate Manner only when it is contextually relevant (Slobin 1996a, b). By contrast, speakers of S-languages like English tend to lexicalise both Manner and Path of motion information with equal frequency, the reason for this being that S-languages typically require these two semantic components of motion to be realised morphosyntactically.

Talmy’s (1985) generalisation, it must be noted, has been challenged by subsequent research; as Aske (1989) observed, in the case of English versus Spanish (and, in fact, in the case of English vs. all Romance languages as well as Greek), Talmy’s proposed typology must be circumscribed to telic motion expressions, namely, lexicalisations of actions involving a change of location (also known as ‘bounded’ motion events; cf. Vendler 1967). Indeed, when the motion event lexicalised is an atelic one, no typological contrast between English and Spanish is observed; for instance:

**Table:** 

(3) *Boris* [F]	*walked* [Mo + Ma]	*around* [P]	*the lake* [G]	(English)

**Table:** 

(4) *Boris* [F]	*caminó* [Mo + Ma]	*alrededor* [P]	*del lago* [G]	(Spanish)
‘Boris	walked	around	(of) the lake’	

In Example 3, the Path-phrase *around the lake* does not predicate a change of location because neither the initial nor the final state of the moving Figure is indicated. In other words, the event is ‘unbounded’ (i.e. Boris could have walked around the lake in perpetuity). In these cases, Spanish does not differ from English in how the motion event is lexicalised, as illustrated by Example 4. By contrast, Spanish and English do exhibit a typological contrast when the motion event being expressed is bounded; this is because Spanish telic events are constructed by using Path verbs such as *entrar* ‘enter’, *cruzar* ‘cross’ or *subir* ‘ascend’, and not by attaching a prepositional Path phrase to a Manner verb. This explains why in Examples 1 and 2 Spanish and English differ in how they lexicalise motion but no difference arises in Examples 3 and 4.

To sum up, English and Spanish differ in how telic motion expressions are encoded: whereas Spanish uses change-of-location verbs to express Path and confines Manner information to an optional adjunct complement, English shows a strong tendency to encode both components: i.e., Manner in the main verb and Path in a satellite or verb particle. It is important to note, however, that both languages are able to use both lexicalisation patterns, although each language is typically associated with one of them. In fact, English has many verbs which encode Path information (e.g. *exit, enter, ascend, descend, cross, turn*). However, in the domain of telic motion, the *preferred* lexicalisation pattern of English is different from the *preferred* lexicalisation pattern of Spanish. This has been confirmed by several studies, including Gennari et al. (2002), Slobin (1996a, b) and Ozcaliskan and Slobin (1999).

### Linguistic Relativity in the Motion Domain

The fact that languages differ in the way they encode different domains of experience does not entail that speakers of different languages conceptualise these domains differently. To explore whether speakers of V- and S-languages conceptualise bounded motion events differently from one another, non-verbal experiments need to be conducted.

Two main methods have been used to test the linguistic relativity hypothesis in the domain of Manner and Path of motion, both of them involving triads of pictures or video-clips (i.e. a target and two alternates). One method relies on *similarity judgments*, which require participants to make a binary choice between two alternates by selecting the one which is perceived as being closer in resemblance to the target. A triad typically presents [Manner α + Path α] in the target, [Manner β + Path α] in the same-Path alternate and [Manner α + Path β] in the same-Manner alternate. Because Manner is hypothesised to be less cognitively salient for V-language (e.g. Spanish) speakers than for S-language (e.g. English) speakers, the task-specific prediction is that, on average, the former should prefer alternates which display the same Path as the given target, rather than alternates with the same Manner.

Studies using similarity judgments have produced conflicting results; the bulk of the evidence, however, supports the view that linguistic and conceptual representations are not causally related. Negative evidence for linguistic relativity in the domain of Manner and Path of motion was found in Bohnemeyer et al. (2001, 2004), Gennari et al. (2002), Papafragou et al. (2002), Pourcel (2005: ch6), Cardini (2010), Papafragou and Selimis (2010; Experiments 2 and 3). Positive evidence, in turn, was found in Finkbeiner et al. (2002), Hohenstein (2005), Papafragou and Selimis (2010; Experiment 1), Czechowska and Ewert (2011), as well as in Kersten et al. (2010), which did not employ similarity judgments but a combination of object-sorting and event-sorting tasks.

The other method used to test the linguistic relativity hypothesis in this domain is *recognition memory*. This task is typically conducted in two sessions. The first part of the experiment requires participants to watch a series of target clips which are played one at a time. In the second part, participants are shown, in a random order, the clips viewed in the first session along with their alternates, and are asked to select only those clips which were shown to them during the first session. The task-specific prediction here is that V-language (e.g. Spanish) speakers would make more errors than S-language (e.g. English) speakers with alternates that have the same Path as the target but that vary in Manner. Negative evidence from memory recognition was found in Gennari et al. (2002) and Papafragou et al. (2002) whereas positive evidence was, to our knowledge, only reported in Filipović (2011).

Similarity judgments and memory recognition require participants to assess the degree to which a controlled set of stimuli have common or distinctive properties. The studies reviewed above, in this sense, provide direct evidence about the relative salience of Manner versus Path in participants’ conceptualisations of bounded motion events (Gennari et al. 2002). A different way to approach the linguistic relativity hypothesis in this domain is to ask whether differences in motion event encoding are related to differences in attention allocation or event inspection. This question has been investigated by Trueswell and Papafragou (2010), who monitored English and Greek native speakers’ eye movements as they inspected animated motion events in preparation for a subsequent recognition task; negative evidence for linguistic relativity was reported, as well as evidence that language affects event inspection when participants use language covertly during event encoding.

A study like Trueswell and Papafragou (2010) strongly suggests that language does not have an enduring effect on how humans inspect motion events, but it is unable to reveal whether there are any language-specific differences in how these events are conceptualised. Though perceptual and conceptual structure are largely inter-dependant, not all aspects of conceptual structure can be read off at the level of perceptual structure (cf. Jackendoff 1983, Smith and Heise 1992). Thus, to test whether English and Spanish speakers *conceptualise* bounded motion events differently, decision-based tasks must be implemented.

## The Study in Context: Previous Shortcomings and a New Direction

As is clear from the previous section, the experimental efforts aimed at investigating whether speakers of V- and S-languages conceptualise bounded motion events differently have produced conflicting results. In this section, I will explore some of the reasons why this might be so. In addition, I will question the evidence obtained in similarity judgment and memory recognition tasks (both negative and positive) and will propose a better suited method to test the linguistic relativity hypothesis in the motion domain.

### When is a Non-verbal Task Non-verbal?

A non-verbal task is a language-free experiment, in which participants are engaged in activities which do not involve the production or comprehension of language (e.g. visual perception, categorisation, memory recognition). However, getting an experiment to be actually non-verbal is not a straightforward matter; the main issue is that participants often make use of covert language in order to make sense of  the stimuli presented to them (cf. Papafragou et al. 2002; Boroditsky et al. 2003). As this is a relatively common phenomenon, it becomes difficult to determine, when significant cross-group differences are found, whether these differences are evidence for linguistic relativity or whether they simply corroborate the established fact that linguistic labels, when generated during a given task, can influence participant’s performance (e.g. Carmichael et al. 1932; Bower et al. 1975; Gennari et al. 2002).

The absence of mechanisms to experimentally distinguish linguistic relativity effects (which are hypothesised to persist in situations in which language is neither being used overtly nor covertly) from transient effects of language on non-verbal cognition has made it difficult for researchers to infer definite conclusions from the evidence available. Hohenstein (2005: 420), for instance, reports a language-specific effect on non-verbal performance; however, towards the end of her study, she acknowledges that this result may be due to participants using language covertly during the task:

Another possible explanation of why English and Spanish speakers appear different from each other in the nonlinguistic task is that they are using a verbal encoding of the events to process similarities in the scenes. In other words, participants could be saying to themselves “he’s marching out the gate” while they watch a video. They could then be using this information, and the saliency of the verb in particular, to choose the matching alternate video, rendering the task linguistic. Indeed, in this study the children were not prevented from verbally encoding the video materials while watching the stimuli.

In the domain of Manner and Path of motion, in fact, all of the studies which have claimed to find linguistic relativity effects have also acknowledged—except for Czechowska and Ewert (2011)—that these effects could be the consequence of participants engaging in inner speech during the task (cf. Finkbeiner et al. 2002: 456; Filipović 2011: 482; Kersten et al. 2010: 649). Following Gennari et al. (2002), I will refer to these transient effects of language on non-verbal behaviour, which are observed to occur only when experience is linguistically mediated, as ‘language-as-strategy’ effects.

I believe that evidence for linguistic relativity can be obtained only by using a dual-task paradigm, namely, a standard non-verbal task plus a shadowing or verbal interference task (e.g. counting aloud, repeating pseudo words, etc.). The rationale behind this belief is as follows: linguistic relativity effects are hypothesised to be deep-seated cognitive biases acquired through the habitual use of language and, in this regard, should be impervious to a verbal interference manipulation; by contrast, language-as-strategy effects, since they are the direct consequence of participants using language covertly during a behavioural task should disappear under verbal interference.

Of all the studies conducted so far aimed at exploring whether the conceptualisation of Path and Manner of motion follows language-congruent patterns (see “Linguistic Relativity in the Motion Domain” section), only two have used a dual-task paradigm, namely Gennari et al. (2002; ‘Shadow’ condition) and Cardini (2010). These studies found that Spanish and Italian speakers supply Manner information less frequently than English speakers when talking about bounded motion events. In addition, Gennari et al. (2002; ‘Naming First’ condition) found that when participants are asked to perform similarity judgements for motion events immediately after having described the events, the linguistic labelling affected their choices. However, under verbal interference conditions, neither Gennari et al. (2002) nor Cardini (2010) found language-specific effects on performance.

This is consistent with a growing number of studies showing that when participants are placed under conditions of verbal interference, cross-group differences disappear. For example, Winawer et al. (2007) found that when English- and Russian-speaking participants were engaged in a shadowing task, language-specific effects on colour discrimination did not arise (see also Roberson and Davidoff 2000; Pilling et al. 2003; Wiggett and Davies 2008). More recently, Athanasopoulos and Bylund (2013) found that differences in grammatical aspect encoding between English and Swedish had an effect on participants’ encoding of motion endpoints in memory; this difference, however, was shown to be neutralised under verbal interference conditions. Taken together, these results suggest that the cross-group differences reported in non-verbal studies such as Finkbeiner et al. (2002) and Hohenstein (2005) are most likely to be evidence for the language-as-strategy account.^3^

A potential problem associated with using a dual-task paradigm needs to be considered, however. A verbal interference task typically involves participants repeating numbers or nonsense words without interruption during a non-verbal task; what this appears to do is to saturate the language faculty and, as a result, prevent participants from solving the task with the aid of covert language. If it could be shown that the verbal interference task suppresses only the language faculty whereas all the other cognitive functions needed to solve the non-verbal experiment remain unaffected by it, then one could safely conclude that a dual-task design is a ‘clean’ method, in the sense that it enables researchers to control for one specific factor, that is, the recruitment of linguistic codes during task performance, and to study how the cognitive system behaves when this factor is taken out of the experimental equation.

It is not clear, however, that a verbal interference manipulation affects participants only by preventing them from recruiting linguistic codes during task performance. For example, research indicates that the cognitive impairments associated with using a mobile phone (either handheld or hands-free) while driving can be as profound as those associated with driving while drunk (Langer et al. 2005; Strayer et al. 2006). Therefore, if linguistic relativity effects are not observed under verbal interference conditions, two interpretations are possible. The first is that these effects simply do not exist; once participants are prevented from relying on internal language during the task, cross-group behavioural differences are no longer observed. The other option is that, because the dual-task paradigm imposes a high processing load on the cognitive system, the evidence for linguistic relativity is swept away; according to this interpretation, cross-group differences disappear not because linguistic codes are prevented from being recruited, but due to the fact that the normal functioning of the system is impaired. The results obtained in the present study, as will be discussed in “Results and Discussion” section, provide evidence in favour of the former interpretation.

### Choosing the Type of Task

As discussed in “Linguistic Relativity in the Motion Domain” section, two methods have traditionally been used to test the existence of linguistic relativity effects in the domain of Manner and Path of motion: i.e. recognition memory tasks and similarity judgments. In what follows, I will discuss some of the problems associated with these two methods and explain why they cannot be fully trusted, even if combined with a verbal interference manipulation.

#### Recognition Memory Tasks (or Blind Testing)

Recognition memory, because it is believed to be a window into the mind’s non-linguistic realm, has been embraced by many as a valid experimental method for testing the linguistic relativity hypothesis in the domain of motion (e.g. Gennari et al. 2002; Papafragou et al. 2002; Filipović 2011). However, this method is not without problems. This type of task, in order to be empirically informative, needs to generate a sufficient number of errors. This means that, on the one hand, it cannot be too easy; were this the case, the number of errors would just be too low and the task therefore uninformative. On the other hand, the task cannot be too difficult; were this the case, participants –regardless of the linguistic group they may belong to– would inevitably produce an extremely high (and similar) number of errors, thus preventing language group effects from surfacing (assuming that these effects exist).

Determining the adequate degree of difficulty for the memory task is not a straightforward business, however; many factors can have an impact on a task’s perceived difficulty, including the number of experimental items used and the duration of the distracting activity between the viewing of the targets and the viewing of the targets and alternates together. As Cardini (2010: 1447) notes, it is very hard (if possible at all) to know in advance what the most adequate values for each of these variables are. Given this, it is not surprising that studies have failed to find an effect of language on memory for Path and Manner of motion even in circumstances in which participants were asked to describe the target events (and thus perform linguistic encoding overtly) prior to the memory task (cf. Gennari et al. 2002; Papafragou et al. 2002).^4^

#### Similarity Judgment Tasks (Testing Determinism?)

Similarity judgments involve participants facing a binary choice: after being presented with a target clip displaying a motion event, they need to decide which of the two alternate clips (one in which Manner varies while Path remains the same and the other in which Path varies but Manner does not) is more similar to the target clip. The problem with this task is that it relies on the assumption that Path and Manner salience are evenly distributed in cognition; in other words, that participants, unless they have a language-specific cognitive bias, should not exhibit a differential degree of cognitive salience for either Path or Manner. If this assumption was correct and linguistic relativity effects existed, the task should technically work: speakers of S-languages (e.g. English) would choose the same-Path (or different-Manner) alternate in approximately 50% of the triads, whereas speakers of V-languages (e.g. Spanish) will make it their preferred choice. This assumption is not supported by the evidence available, however.

For example, the results obtained in Gennari et al. (2002) and Cardini (2010) show that participants, regardless of their linguistic background, have a preference for same-Path alternates, which suggests that Path is more cognitively salient than Manner. This is not at all surprising; ‘directionality in human motion’, as Pourcel (2004: 510) notes, ‘may be understood to represent the very goal of motion—at least in typical cases. Such a possibility would mean that the Path dimension overrides the Manner dimension in the cognitive appreciation of human motion as a general rule’. Pourcel’s observation is in accord with the proposed universal bias to prioritise Goal over Source information in motion events; see Lakusta and Landau (2005) and Lakusta et al. (2007).

Why, then, is a binary-choice task like similarity judgments ill-equipped to detect linguistic relativity effects? Because if participants are biased by universal principles of event cognition to prefer the same-Path over the same-Manner alternate, any hypothetical language-specific cognitive bias is likely to be overridden by these principles. A task like similarity judgments, in this regard, is not designed to test the linguistic relativity hypothesis but, rather, linguistic determinism, a proposal which has long been abandoned in cognitive science.

#### Similarity Ratings: A Promising New Direction

Similarity ratings belong to the same family as similarity judgments; however, while in the latter participants have only a binary choice, in the former they can choose among several points on a graded rating scale, which enables them to give a much more precise similarity measure.

Czechowska and Ewert (2011) is the only study in the domain of Path and Manner of motion which, in addition to binary judgments, also used similarity ratings. This study, as mentioned in “Linguistic Relativity in the Motion Domain” section, reports having found positive evidence for linguistic relativity; this finding, however, should be treated with extreme caution. Not only did Czechowska and Ewert (2011) not employ a verbal interference manipulation, but the stimuli used consisted of pictures instead of videos, which renders its results and conclusions questionable. To test the linguistic relativity hypothesis in the motion domain, participants must attend to real motion events and not, as Cardini (2010) notes, infer motion from static images.

In the previous section, it was argued that if there were subtle differences across language groups in the way motion events are conceptualised, binary judgements would likely not be able to capture this variation. Would similarity ratings do any better? If instead of asking participants to select one alternate on the basis of similarity to the target, they were instructed to assign a similarity rating to [target + alternate 1] as well as one to [target + alternate 2] using a Likert scale, two types of information would be made available: (1) which of the two target-alternate combinations received, on average, the higher rating, and (2) the difference between the two target-alternate combinations’ mean ratings. (1) should reveal which dimension of motion, Path or Manner, was conceptualised as being the more salient in that particular triad, a result which should be language-independent. (2), in turn, should tell us how much more salient one dimension was deemed to be in relation to the other across language groups –that is, whether or not linguistic relativity effects were observed. Undoubtedly, the subtler the linguistic relativity effects, the smaller the chances of bringing them to the surface. What is clear is that a similarity rating task has at least a good chance of finding differences in motion conceptualisation across different language groups. For this reason, similarity ratings (and not binary judgments) were used in the present study.

### Summary

None of the studies claiming to find linguistic relativity effects have employed a verbal interference manipulation; it is therefore likely that, in these studies, performance was mediated by language. Moreover, in the studies where a verbal interference task was included, linguistic relativity effects were not observed (e.g. Gennari et al. 2002 and Cardini 2010), which suggests that the habitual use of language does not have an enduring effect on non-verbal cognition. The latter studies, however, relied on experimental methods (i.e. binary judgments and memory recognition) which, had linguistic relativity effects existed, would have likely failed to detect them. As a result, neither the ‘positive’ nor the ‘negative’ evidence for linguistic relativity in the domain of motion conceptualisation can be regarded as conclusive.

## The Present Study

The present study explores whether different lexicalisation patterns in English and Spanish predict how speakers of these languages perform in a non-verbal experiment. The overarching research question that guided this investigation is as follows: *given the fact that English speakers indicate Manner information with greater frequency than Spanish speakers when talking about bounded motion events, is it the case that Manner is less cognitively salient for Spanish than for English speakers?*

In order to answer this question, two experiments were conducted: first, a similarity rating task, in which participants rated sequences of two motion events on the basis of how similar to each other these events were perceived to be; second, a verbal elicitation task, in which participants were asked to verbally describe the stimulus material used in the rating task. The experiments were carried out in this order for a reason: if participants had been asked to describe the target items first, this initial coding could have had an influence on how participants behaved in the subsequent similarity rating task, thus contaminating what was supposed to be a non-verbal experiment. The similarity rating task, therefore, preceded the verbal elicitation task.

This study shares the same rationale as previous studies aimed at testing the linguistic relativity hypothesis in the motion domain. The rating task was designed to explore whether English and Spanish speakers follow language-congruent patterns when asked to make similarity judgments of sequences of bounded motion events. The verbal elicitation task, in turn, was a validation test; that is, it was aimed at showing that the motion events used in the rating task reproduced Talmy’s (1985) typology when participants were asked to describe them verbally. Thus, if significant cross-group differences were observed to occur in the similarity rating task, these differences could then be related to participants’ verbal performance in the elicitation task. However, if no significant cross-group differences were revealed by the similarity rating task, but differences in verbal performance emerged in the elicitation task, then it could be concluded that linguistic structure is not a predictor of non-verbal behaviour. The results obtained in this study are in keeping with this latter scenario.

## Experiment I

Experiment I was designed by paying special attention to the lessons learned from previous attempts to test the linguistic relativity hypothesis. First, the classic binary judgment task was replaced by similarity ratings; participants viewed sequences of two motion events and were asked to rate them using a 7-point Likert scale, ranging from [0] at the lowest similarity point to [6] at the highest. Second, the task was combined with a verbal interference manipulation, to make sure that the results obtained, if positive, could be confidently interpreted as linguistic relativity effects. Finally, unlike previous studies, a control condition was included so that cross-group differences, if found, could be uncontroversially explained by reference to the typological contrast observed between the two languages studied.

### Method

#### Participants

18 monolingual speakers of English and 18 monolingual speakers of Spanish were tested. 2 participants per group had to be discarded for either failing to pass the attentional controls (see “Non-critical Stimuli” section) or giving highly inconsistent responses. For the purpose of this study, a monolingual speaker was defined as one who spoke fluently only their native tongue and had never lived for longer than 6 months in a country where a different language from their mother tongue was spoken.

The English speakers were all students at the University of Cambridge (mean age = 23) and were tested in a quiet room provided by the university. The Spanish-speaking group, in turn, were composed of tertiary-level students and graduates in full-time employment residing in Buenos Aires, Argentina (mean age = 25.5), and were tested in similar conditions to the English speakers.

#### Materials

##### Stimuli

30 experimental items (or dyads) were used, each of them consisting of a sequence of two video clips, i.e.: a model and an alternate clip. The clips, 45 in total, were filmed with a DSC-H20 Sony digital video camera and showed a person or an object performing a motion event in a real-life environment. The average duration of a clip was of 3.5 s. Though some clips appeared in more than one dyad, each dyad displayed a distinct combination of clips.

**Fig. 1:**
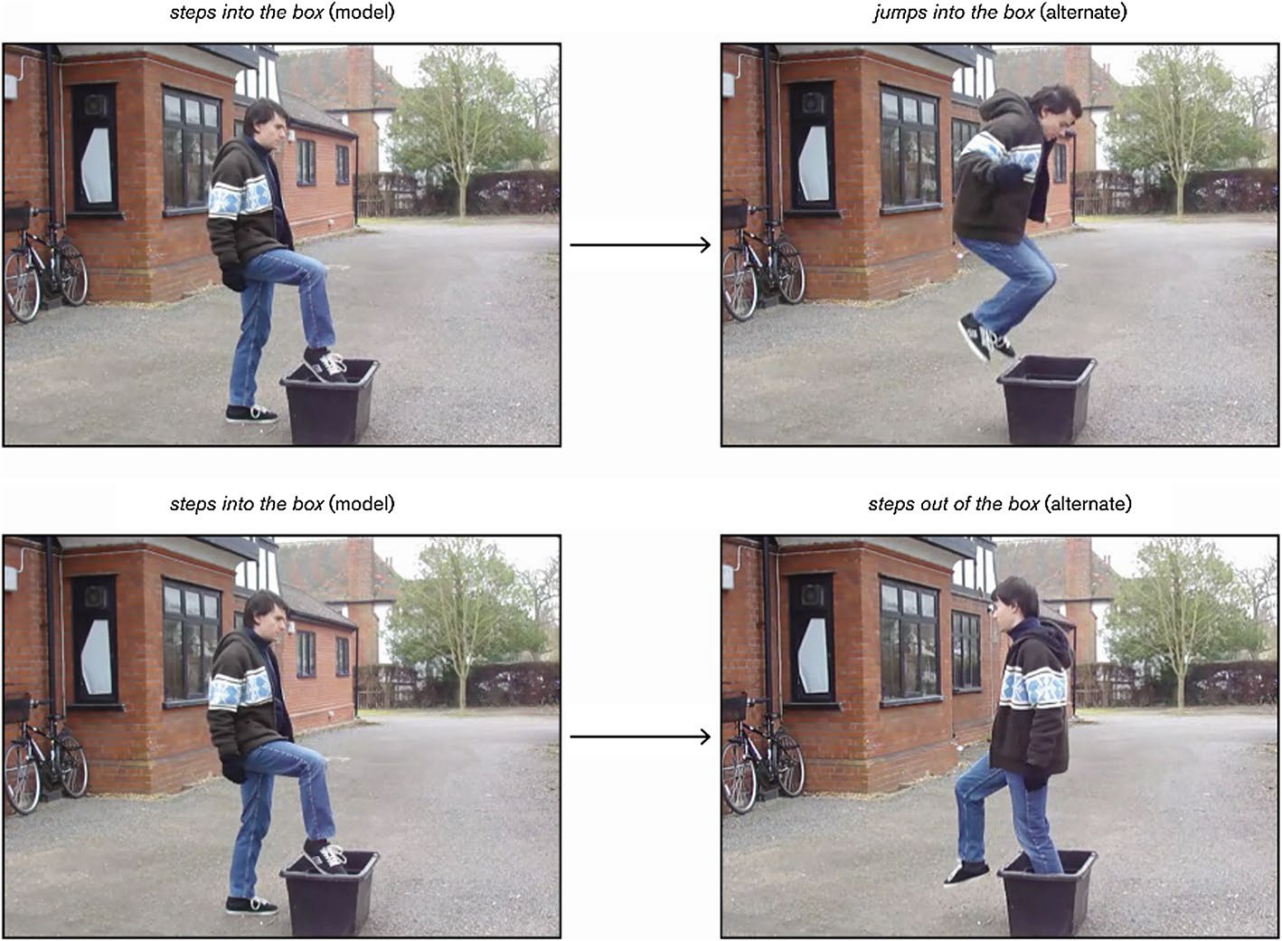
A set of sister dyads, the first from the different-Manner (DM) condition and the second from the different-Path (DP)

*Critical Stimuli* 46.6% of the trials used critical stimuli, which consisted of 7 different-Manner (DM) and 7 different-Path (DP) items. Every dyad in the DM condition had a ‘sister’ in the DP condition. A set of sister dyads, as illustrated in Fig. 1, shared the same model clip yet differed in terms of the alternate clip: whereas in the DM dyad the alternate clip was the same as the model save for the fact that the Figure’s Manner changed, in the DP dyad the alternate clip was the same as the model except for the fact that the Figure’s Path changed.

The motion events in the DM and DP conditions were bounded and, therefore, were expected to trigger a typological contrast between English and Spanish (see “The Typology of Motion Expressions: The Case of English Versus Spanish” section). The clips displayed a total of 7 Manners (i.e*. run, walk, step, jump, climb, bounce, roll*), 6 Paths (i.e. *into, out [of], down, up, onto, off*), 2 Figures (i.e. *man, ball*) and 5 Ground objects (i.e. *house, plastic box, stairs, chair, cardboard box*). The DM and DP items used are listed in Table 1.

**Table 1:** Items in the DM and DP conditions

No.	Model clip	Alternate clip	Ground object
*DM condition*			
1	**runs into**	**walks into**	house
	entra (corriendo)	entra (caminando)	
2	**walks out**	**runs out**	
	sale (caminando)	sale (corriendo)	
3	**steps into**	**jumps into**	plastic box
	entra (caminando)	entra (de un salto)	
4	**runs down**	**walks down**	stairs
	baja (corriendo)	baja (caminando)	
5	**walks up**	**runs up**	
	sube (caminando)	sube (corriendo)	
6	**jumps onto**	**climbs onto**	chair
	sube (de un salto)	sube (trepando)	
7	**bounces into**	**rolls into**	cardboard box
	entra (picando)	entra (rodando)	
*DP condition*			
1	**walks out**	**walks into**	house
	sale (caminando)	entra (caminando)	
2	**runs into**	**runs out**	
	entra (corriendo)	sale (corriendo)	
3	**steps out of**	**steps into**	plastic box
	sale (caminando)	entra (caminando)	
4	**walks up**	**walks down**	stairs
	sube (caminando)	baja (caminando)	
5	**runs down**	**runs up**	
	baja (corriendo)	sube (corriendo)	
6	**climbs off**	**climbs onto**	chair
	baja (agarrándose)	sube (trepando)	
7	**rolls out of**	**rolls into**	cardboard box
	sale (rodando)	entra (rodando)	

**Fig. 2:**
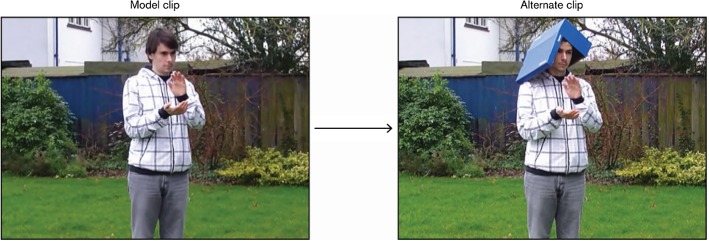
An experimental item from the CD condition

*Non-critical Stimuli* 53.3% of the trials used non-critical stimuli, which consisted of 7 controls (which also served as distractors), 7 ‘regulators’ and 2 attentional controls. Just like the DM and DP conditions, the control/distractor (CD) condition was composed of dyads in which the alternate clip differed from the model clip; however, this condition was distinct in two respects. First, the events depicted in each clip were not bounded and, therefore, did not trigger a typological contrast between English and Spanish. Additionally, in these items, the dimension which underwent change was not motion-related (see Fig. 2). A list with the CD items used is provided in “Appendix 1”, Table 2.

**Fig. 3:**
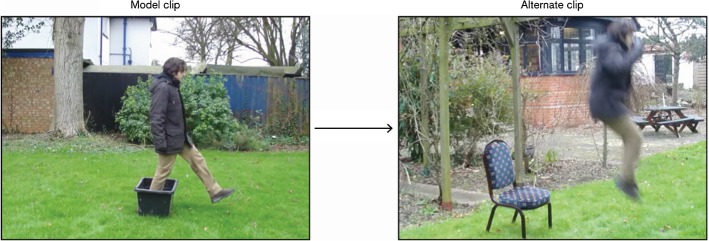
A regulator item

The regulators were designed with the intention of minimising idiosyncratic uses of the scale. The model and alternate clips present in each regulator differed from one another in more than one dimension and, therefore, were visibly less similar than the dyads in the other conditions. Some of the regulators differed on two dimensions (e.g. Path and Ground underwent change whereas Manner and Figure remained the same) and some on three (e.g. Path, Manner and Ground underwent change whereas only the Figure remained the same); an example is provided in Fig. 3.

By including these items among the stimulus material, an objective rating criterion was implicitly established: a dyad in which model and alternate differed on two or three dimensions should be given a lower similarity rating than a dyad in which only one dimension underwent change. Thus, the scope of individual variation was reduced (but not nullified): a regulator was expected to be rated somewhere between [0] and [2], whereas a DM, DP or CD item between [3] and [5]. A list with all the regulators used is provided in “Appendix 1”, Table 3.

**Fig. 4:**
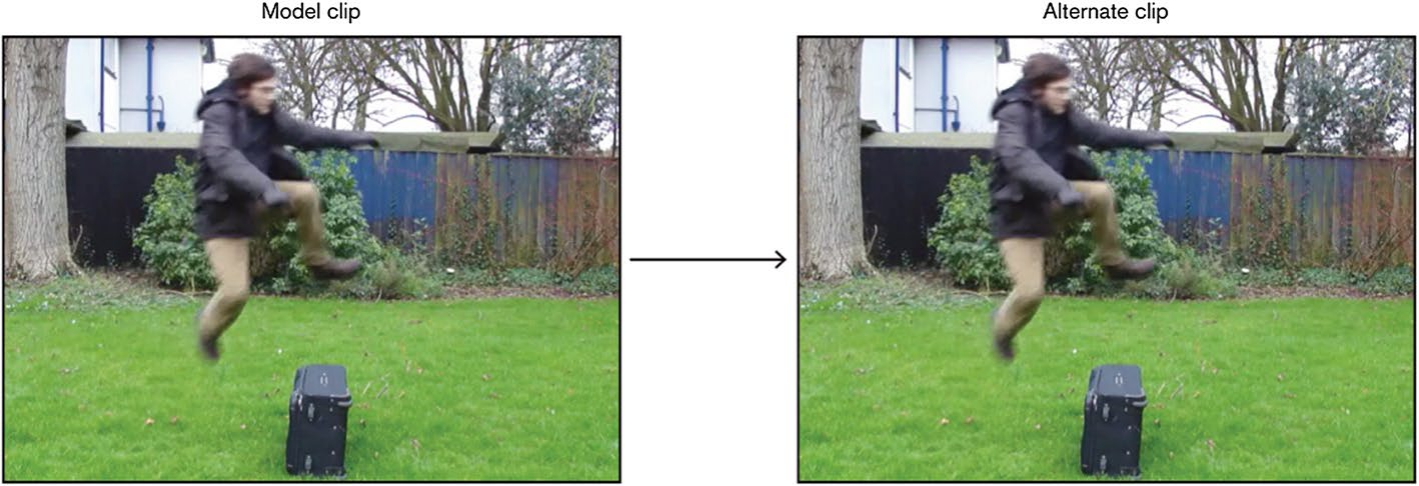
An attentional control

A rating of [6] meant ‘completely similar’ and was expected to be assigned only to the attentional controls. The purpose of these items, which consisted of two identical clips, was to make sure that participants were actually paying attention and not giving random responses. Participants who rated attentional controls with a number lower than [5] were discarded. An example of attentional control is given in Fig. 4.

##### Shadowing Task

The shadowing or verbal interference task consisted of a random sequence of 20 CVC nonsense syllables, separated from each other by 2-second blank intervals. Participants listened to the nonsense syllables via a pair of Sony MDR570LPB headphones and repeated them back whilst performing the similarity rating task. Two versions of the same shadowing task were created, one compatible with the English sound system and the other with the Spanish. The English nonsense syllables were taken from Boothroyd and Nittrouer (1988) whereas the Spanish ones were especially designed for this experiment. All the nonsense syllables were recorded by the experimenter using a Zoom H4n Handy Recorder.

#### Procedure

The experiment was conducted in a quiet room using Microsoft Office Power Point 2013 on a Samsung Series 5 portable PC laptop. Subjects were tested in their native language and given a monetary reward for their participation. The experiment took 6 min and 37 s to be completed and included a short training task at the beginning.

Subjects sat opposite a laptop computer screen displaying a 7-point Likert scale. Participants were told that the task involved rating sequences of two video clips on the basis of how similar to one another these two clips were perceived to be. They were instructed to do this as soon as the second clip in the sequence finished playing.^5^

**Fig. 5:**
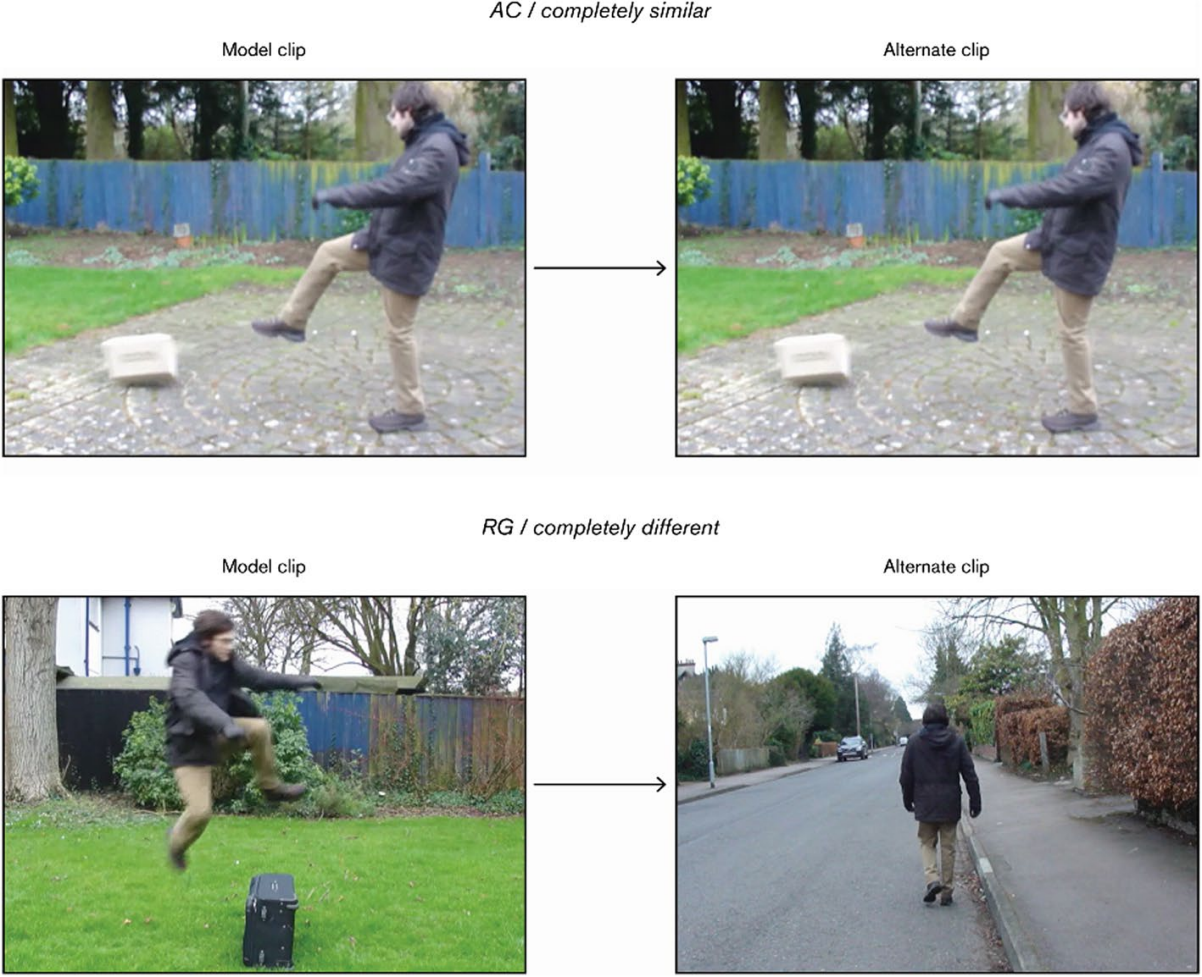
Attentional control (AC) and regulator (RG) used to calibrate participants to the scale’s extremes

As to the scale, participants were told that similarity increased from left to right, as the numbers increased. They were instructed that a rating of [0] was appropriate for those sequences in which the clips played were *completely different* from one another whereas a rating of [6] was meant for those which were *completely similar* or *identical*. In order to make it clear what *completely different* and *completely similar* meant in the context of the experiment, an example of a [0] and a [6] item (a regulator and an attentional control respectively) was shown to the participants prior to starting the task (see Fig. 5). The aim of this was to calibrate participants to the scale.

The procedure was simple. The experimenter initiated the verbal interference manipulation first; once the subject had repeated nonsense syllables for 10 s, the rating task was started. Participants always saw two clips played in sequence and, subsequently, a rating scale, which stayed on the screen for only 3.5 s. Participants, who kept repeating nonsense syllables until the end of the task, rated the sequences of clips by pointing to the scale with a rubber pen. Each rating given was recorded by the experimenter on an answer sheet.

Since some clips featured in more than one dyad, it was reasonable to expect priming effects. For instance, the second time that participants saw the clip *a man walks into the house*, which was present in both DM-1 and DP-1 (see Table 1), their previous experience with it could have exerted an influence on the way in which this clip was conceptualised. Whether this could have had an undesirable effect on the actual rating task is uncertain. To control for this eventuality, items were presented in two different orders. To one half of the participants, items were presented according to order A, whereas order B, which was order A reversed, was used with the other half of the participants. Had priming effects of the sort described above existed, this technique would have numerically cancelled them.

### Task-Specific Prediction

The linguistic relativity hypothesis in the domain of motion predicts that the dimension of Manner should be less cognitively salient for Spanish than for English speakers, as the former do not habitually specify Manner information when talking about bounded motion events. Conversely, since Path information must be obligatorily specified in both English and Spanish, there are no reasons, at least as far as the linguistic relativity hypothesis is concerned, to expect significant differences in Path conceptualisation between the two groups tested.

Thus, according to this hypothesis, the Spanish speakers should, on average, give the DM (different-Manner) items a higher similarity rating than the English speakers whereas the DP (different-Path) items should not trigger cross-group differences. However, as the two groups might exhibit different baseline response biases, the most accurate prediction that can be articulated is as follows: *the difference between the Spanish speakers’ DM and DP rating means should be significantly larger than the difference between the English speakers’ DM and DP rating means.*

### Results and Discussion

**Fig. 6:**
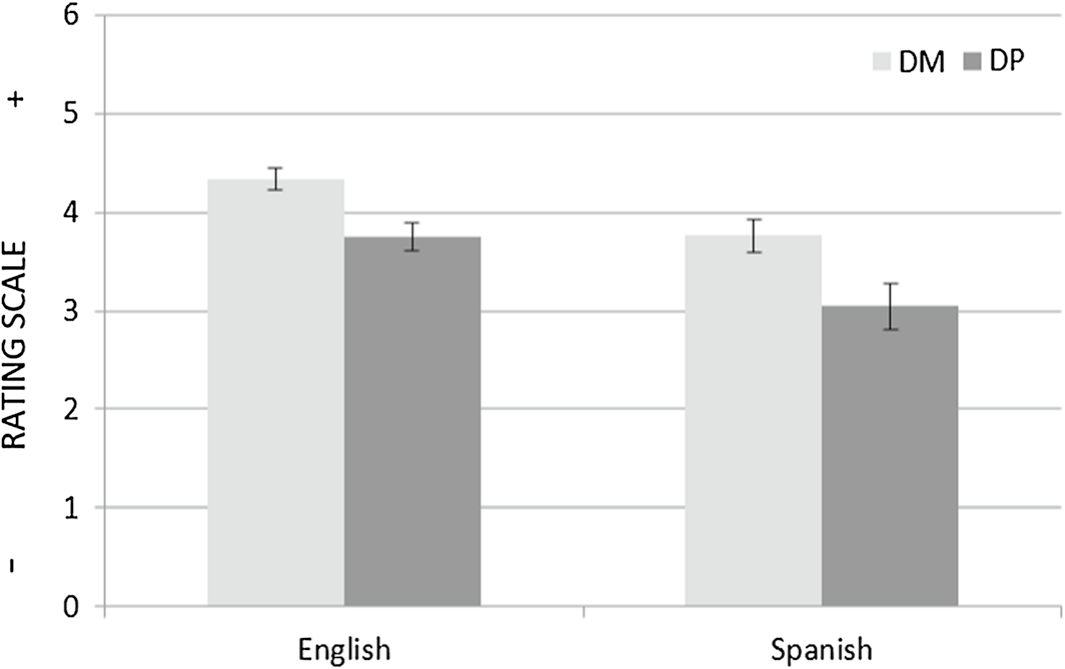
Mean ratings with Standard Error Bars for the DM and DP conditions

On average, the English speakers rated both DM and DP items higher on the scale than the Spanish speakers, i.e.: M_DM_ = 4.34 (English) versus M_DM_ = 3.76 (Spanish); M_DP_ = 3.75 (English) versus M_DP_ = 3.04 (Spanish) (see Fig. 6). To test the task’s prediction, a 2 × 2 mixed ANOVA with group (English vs. Spanish) as a between-subjects factor and condition (DM, DP) as a within-subjects factor was run. The test revealed a main effect of group (F (1, 30) = 8.345, *p* < 0.05); however, there was no significant interaction between group and condition (F (1, 30) = 0.593, *p* > 0.05, η_p_^2^ = 0.019). The lack of interaction indicates that the difference between the Spanish speakers’ DM and DP rating means was not significantly larger than the difference between the English speakers’ DM and DP rating means. This result does not support the prediction made in “Task-Specific Prediction” section.

**Fig. 7:**
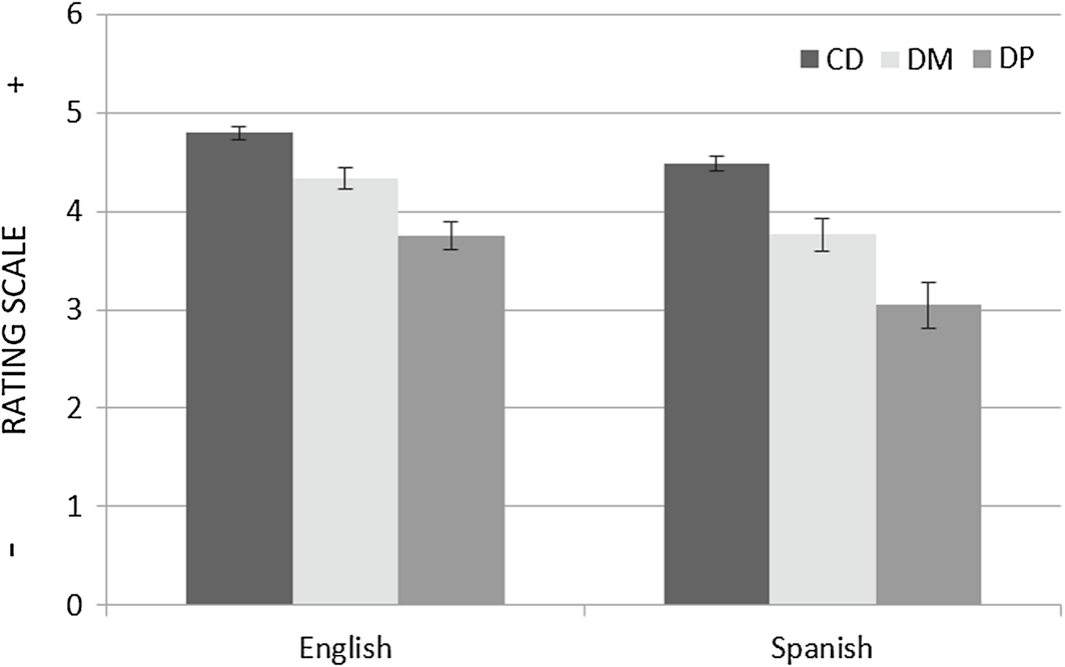
Mean ratings with Standard Error Bars for the DM, DP and CD conditions

The question that remains to be answered is why the mean ratings of the two groups tested significantly differed in both the DM and DP conditions. The most likely explanation is that the English and Spanish speakers had different rating baselines. In order to discard, however, the possibility of language having had any influence on participants’ ratings, an ANOVA test with the mean rating for the CD (control/distractor) condition as dependant variable and group (English vs. Spanish) as a between-subjects factor was run. The CD condition, as explained in “Non-critical Stimuli” section, was specifically designed not to trigger cross-group variation; the ANOVA test, however, revealed that the English speakers rated CD items higher on the scale than the Spanish speakers and that this difference was statistically significant (F (1, 30) = 8.892, *p* < 0.05) (see Fig. 7). Since the same effect of group observed in the DM and DP conditions also surfaced in the CD condition, this effect cannot be explained by evoking Talmy’s (1985) typological contrast between V- and S-languages.

In order to determine whether there were still significant differences between groups once the rating means for the DM and DP conditions were adjusted for the difference found in the CD condition, two ANCOVA tests were conducted: one with the mean rating for the DM condition as dependant variable and the other with the mean rating for the DP condition; in both cases, the mean rating for the CD (control) condition was used as co-variant and group was the between-subjects factor. After the adjustment, the effect of group reported above completely disappeared (F_DM_ (1, 29) = 2.237, *p* > 0.05; F_DP_ (1, 29) = 1.113, *p* > 0.05). The results obtained in this experiment, therefore, do not corroborate the linguistic relativity hypothesis.

In “When is a Non-verbal Task Non-verbal?” section, the issue of whether a verbal interference task has the capacity to interfere with non-verbal cognitive processes was raised. A first possibility is that it suppresses only the language faculty, thus preventing participants from recruiting linguistic codes during the task. If this is what in fact occurs, linguistic relativity effects, had they existed, should not have passed undetected. The other possibility is that the verbal interference interferes with other cognitive processes (in addition those specific to language) and thus bleaches any trace of linguistic relativity. The results obtained in the present study offer support for the former scenario.

As discussed in “Similarity Judgment Tasks (Testing Determinism?)” section, the Path dimension seems to override the Manner dimension in the cognitive appreciation of human motion as a general rule (cf. Pourcel 2004, 2005). Studies such as Gennari et al. (2002) and Cardini (2010), which used animated motion events with human Figures, provide good evidence for this i.e.: participants exhibited a preference for choosing same-Path (or different-Manner) over same-Manner (or different-Path) alternates regardless of their linguistic background.

Pourcel (2004, 2005) has speculated that the tendency to prioritise Path (which in telic motion events typically codes the very goal of motion) over Manner may be related to a number of properties present in the event being perceived, including whether the Figure performing the motion is an animate or inanimate entity. She (2005: 247) conjectures, for example, that ‘inanimates, such as objects, may not be perceived as undergoing or initiating intentioned motion with purposeful PATH ENDPOINTS’. According to Pourcel (2004, 2005), this would predict that behavioural tasks in which an inanimate Figure is used may fail to reproduce the Path salience observed in studies like Gennari et al. (2002) and Cardini (2010). The evidence obtained in Zlatev et al. (2010: ch5, study 1) seems to support this prediction: in a similarity judgment task whose stimuli was computer-generated and displayed a tomato as Figure, participants’ choices did not exhibit a Path bias.

**Fig. 8:**
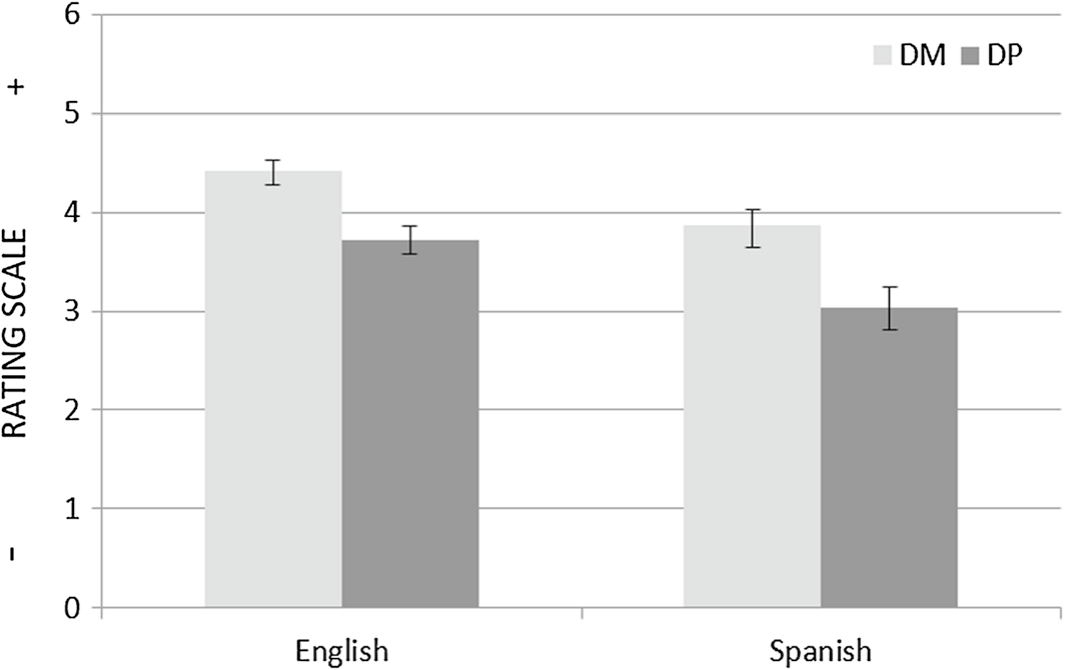
Mean ratings with Standard Error Bars for the DM and DP items (human motion)

In accord with these patterns, in the present experiment participants conceptualised motion events differently according to whether the moving Figure was an animate or an inanimate entity. When the events presented involved human motion, all participants, regardless of their linguistic background, rated DP items, on average, lower on the scale than DM items: for the English group, M_DP_ = 3.72 versus M_DM_ = 4.42; for the Spanish group, M_DP_ = 3.03 versus M_DM_ = 3.86. A 2 × 2 mixed ANOVA revealed a significant within-subjects main effect of condition (F(1, 30) = 89.47, *p* < 0.05) as well as a significant between-subjects main effect of group (F(1, 30) = 8.03, *p* < 0.05). Overall, this indicates that, in the case of dyads involving human motion, Path changes were cognitively more salient than Manner changes (see Fig. 8).

**Fig. 9:**
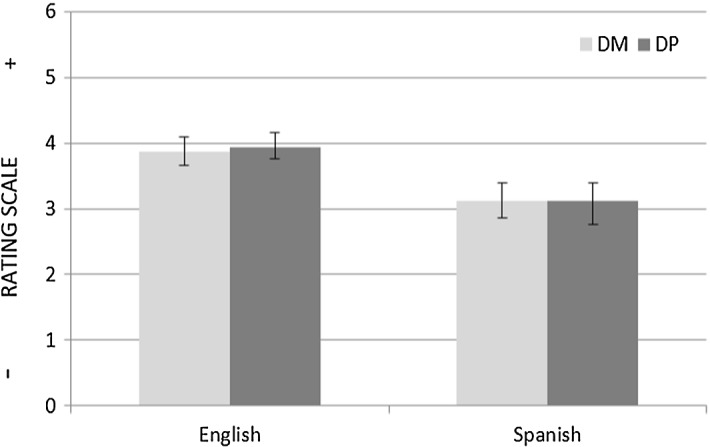
Mean ratings with Standard Error Bars for the DM and DP items (object motion)

**Fig. 10:**
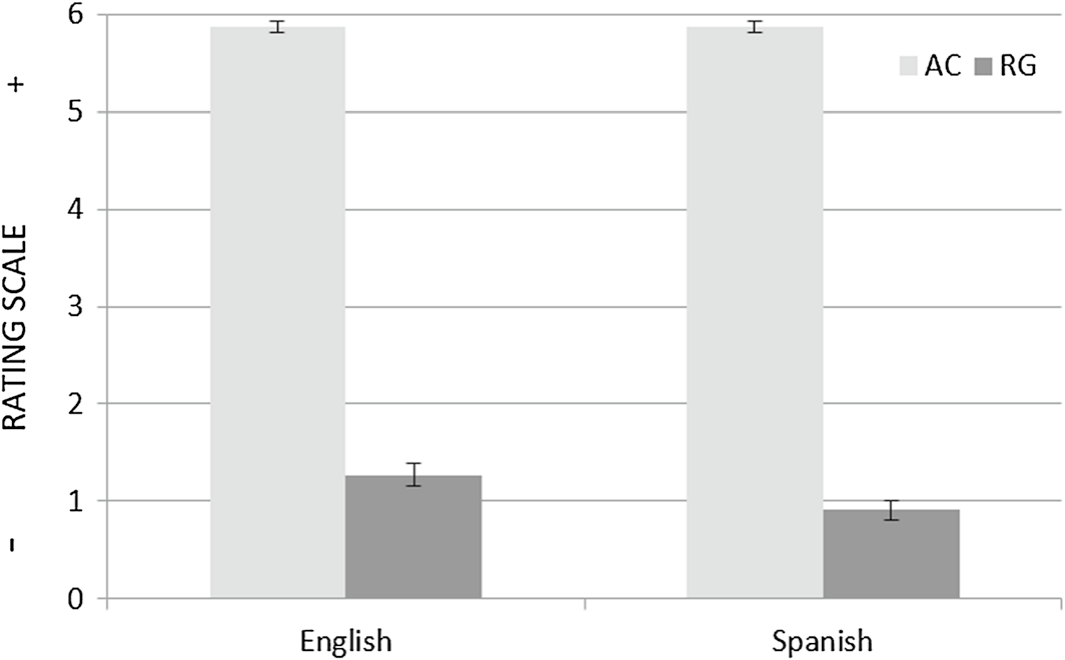
Mean ratings with Standard Error Bars for attentional controls (AC) and regulators (RG)

Among the stimuli used, however, two experimental items displayed a ball as Figure (i.e. DM-7 & DP-7, Table 1).^6^ Notably, participants did not exhibit a Path bias when rating these items (see Fig. 9): for the English group, M_DP_ = 3.94 versus M_DM_ = 3.88; for the Spanish group, M_DP_ = 3.13 versus M_DM_ = 3.13. A 2 × 2 mixed ANOVA showed a significant between-subjects main effect of group (F(1, 30) = 6.27, *p* < 0.05); this time, however, no within-subjects condition effect was detected (F(1, 30) = 0.02, *p* > 0.05). The fact that participants’ similarity judgments were sensitive to the Figure’s animacy provides evidence that the verbal interference manipulation used did not interfere with what are likely to be universal mechanisms of event conceptualisation. Furthermore, participants were able to identify those dyads in which the two clips displayed were identical (attentional controls) and rate them accordingly. Likewise, those dyads in which the clips were very different from each other (regulators) were consistently given low ratings (see Fig. 10).

Previous studies in motion conceptualisation (Gennari et al. 2002; Cardini 2010) and inspection (Trueswell and Papafragou 2010) which adopted a dual-task paradigm provided no evidence of whether the shadowing task used interfered with general cognitive mechanisms of event perception and conceptualisation. In these studies, it is therefore unclear why linguistic relativity effects do not arise: is it because the normal functioning of the cognitive system is being disrupted by the interference task or is it simply because the hypothesised effects do not exist? The results obtained here suggest that non-verbal cognitive processes (at least those involved in motion conceptualisation) are unaffected by the shadowing task. This offers support to the view that the dual-task paradigm (non-verbal task + shadowing task) is a valid method to test the linguistic relativity hypothesis.

## Experiment II

Experiment II consisted of a verbal elicitation task, and was designed to determine whether the stimulus material used in Experiment I, when verbally described, triggered the typological contrast between English and Spanish established in Talmy (1985).

### Method

#### Participants

The participants tested were the same as in Experiment I. Participants completed Experiment I first and only subsequently were engaged in the present task.

#### Materials

The video clips used in the DM (different-Manner) and DP (different-Path) conditions (Experiment I) were also used in this task. In addition, 8 filler clips were included, which were randomly picked from Experiment I’s regulators and CD (control/distractor) items.

#### Procedure

Participants were informed that they would watch a series of video clips but, unlike in the previous task, the clips would be presented one by one as opposed to paired in dyads. After each clip reached completion, participants were asked to tell the experimenter in their respective native languages what had happened (i.e. *English: what’s happened in the clip?*, Spanish: *Qué ha sucedido en el video?*). Participants’ responses were recorded using a Zoom H4n Handy Recorder and subsequently transcribed for analysis. The order of presentation of the stimuli was the same for all participants.

### Results and Discussion

**Fig. 11:**
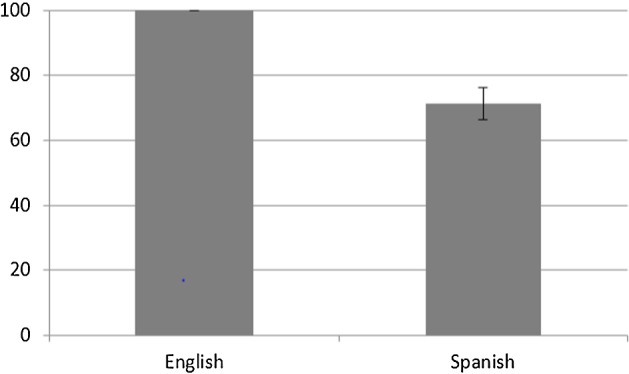
Percentage of utterances with Manner information with Standard Error Bars (%)

Like in Gennari et al. (2002), and in accord with Talmy’s (1985) typology, the results obtained indicate that the English and Spanish speakers differed in terms of the frequency with which Manner information was supplied: whereas the English speakers lexicalised Manner information in 100% of their utterances, the Spanish speakers gave such information in 71.25% of theirs (see Fig. 11). An ANOVA test on the proportion of cases where Manner was specified with group (English vs. Spanish) as a between-subjects factor confirmed that there was a significant main effect of group (F (1, 30) = 33.113, *p* < 0.05, η_p_^2^ = 0.52).

**Fig. 12:**
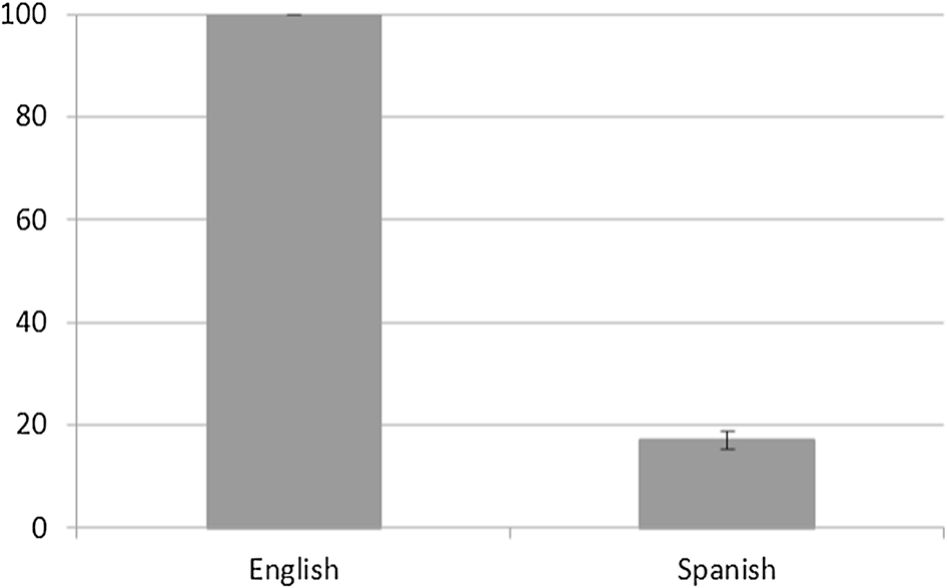
Percentage of utterances with Manner coded in main verb with Standard Error Bars

The English-speaking participants also differed from the Spanish in terms of where in the sentence Manner and Path were coded. Whereas the English speakers coded Manner information in the main verb in 100% of their utterances, the Spanish speakers did so only in 16.98% of theirs (see Fig. 12). The latter group, as expected, preferred to express Path in the main verb (e.g. *subir* ‘ascend’, *entrar ‘*enter’) and supplied Manner information in the form of an optional gerund or adverbial complement at the end of the sentence (e.g. *caminando* ‘walking’, *rápidamente* ‘quickly’). An ANOVA on the proportion of cases where Manner was coded in the main verb with group (English vs. Spanish) as a between-subjects factor showed a significant main effect of group (F (1, 30) = 2410.728, *p* < 0.05, η_p_^2^ = 0.99).^7^

Though the results confirm only what is already well-established in the literature, the fact that the expected linguistic differences between the English and Spanish speakers surfaced so clearly confirms the strength of the stimuli design and indicates that the conceptualisation of motion events does not follow language-congruent patterns^8^: indeed, though the Spanish speakers supplied Manner information less frequently than the English speakers when asked to describe a series of bounded motion events, the two groups of participants, when asked to make similarity judgements of paired configurations of the same events, showed equal sensitivity to the dimension of Manner.

## General Discussion

At the outset of this article, two different views on the relationship between language and thought were introduced. According to the linguistic relativity position, linguistic representations are able to exert an enduring influence upon those cognitive processes required to segment and conceptually represent events. By contrast, according to the universalist position, non-verbal cognitive processes are impervious to linguistic influence; on this view, all humans share the same conceptual structure, but possess different rules mapping conceptual components onto lexical and syntactic frames. In the present study, by comparing non-verbal performance in a similarity rating task to linguistic performance in an elicitation task, I have found evidence that supports the universalist position.

It could be argued that linguistic relativity effects were not found because the typological contrast between the two languages studied is too tightly circumscribed, being observed only in telic motion expressions. In addition, as discussed in “The Typology of Motion Expressions: The Case of English versus Spanish” section, these two languages can exhibit both V- and S-language behaviour, which means that speakers are not forced to use one or other lexicalisation strategy. Is the typological contrast between English and Spanish strong enough so as to render relativity effects? This question is difficult to answer because, so far, and as far as I am aware, no compelling evidence of enduring effects of language on non-verbal cognition has been presented, neither in the domain of motion nor in any other domain.^9^

Lucy (2010), for example, maintains that lexico-grammatical patterns which are used ubiquitously are likely to have the greatest impact on thought. Much of his work has in fact been devoted to showing that speakers of languages with obligatory grammatical number marking and count-mass noun distinction (e.g. English) are more likely to base their similarity judgements of objects on shape than speakers of non-plural marking classifier languages (e.g. Yucatec) who tend to base their judgments on substance properties. It remains unclear, however, whether the pervasiveness and systematicity of a given lexico-grammatical pattern is correlated to the probability of finding linguistic relativity effects. Though it is true that Lucy (Lucy 1992a; Lucy and Gaskins 2001, 2003) reported language-specific effects on object categorisation, these studies do not constitute conclusive evidence for linguistic relativity, the reason for this being simple: English and Yucatec speakers did not perform under verbal interference conditions.

A more likely explanation for why linguistic relativity effects have not been found in the present study (in spite of having carefully designed Experiment I to increase the likelihood of finding them) is that language may exert only a transient influence on thought. This study is in fact a contribution to the emerging picture that language effects on non-verbal performance do not surface when participants are placed under conditions of verbal interference, which indicates that language is unlikely to have an enduring effect on non-verbal cognition.

## Conclusions

This article examined whether the dimension of Manner is less cognitively salient for Spanish than for English speakers, a question motivated by the observation that these two languages typically differ in terms of the frequency with which Manner information is supplied in telic motion expressions. To address this question, English and Spanish native speakers’ performance in a dual-task experiment (similarity ratings + shadowing task) was compared to their linguistic performance in a verbal elicitation task. Though the analysis of the non-verbal data exposed a main effect of group, this effect was shown not to be causally related to the cross-group differences revealed by the verbal elicitation task. Evidence was also provided that shadowing tasks such as the one used in this study do not seem to interfere with the cognitive mechanisms involved in event conceptualisation, offering support to the view that the dual-task paradigm is a valid method to test the linguistic relativity hypothesis in the motion domain.

## Appendix 1

See Tables 2 and 3.

**Table 2:** Items in the control/distractor (CD) condition

No.	Model clip	Alternate clip
1	**jumps**	**(a man with scarf) jumps**
	salta	(un hombre con bufanda) salta
2	**hits the wall**	**(a man with hat) hits the wall**
	golpea la pared	(un hombre con sombrero) golpea la pared
3	**rotates**	**rotates (with a box)**
	rota	rota (con una caja)
4	**claps**	**claps (with a box on his head)**
	aplaude	aplaude (con una caja en la cabeza)
5	**runs around bush**	**(a man with a different coat) runs around bush**
	corre alrededor del árbol	(un hombre con otro saco) corre alrededor del árbol
6	**hugs a tree**	**(a man with a different jacket) hugs a tree**
	abraza el árbol	(un hombre con otra campera) abraza el árbol
7	**runs on the spot**	**(a man with umbrella) runs on the spot**
	corre en el lugar	(un hombre con paraguas) corre en el lugar

**Table 3:** Regulators used in Experiment I

No.	Model clip	Alternate clip
1	Skips across the road	Skips up to the tree
2	Steps out of the box	Jumps down from chair
3	Runs on the spot	Runs up to the wall
4	Skips away from tree	Runs up to the wall
5	Walks across the road	Walks around the box
6	Runs up to the wall	Jumps from table
7	Walks along the street	Waves to camera

## Appendix 2

In this appendix, the verbal descriptions of 3 critical items (cf. “Critical Stimuli” section) produced by three randomly chosen participants from each language group are provided. Regarding the elicitations in Spanish, the criterion to count a token as Manner was that the token could clearly answer the question: *In which Manner did the Figure move?* For instance, the Prepositional Phrase *por el piso* ‘against/along the floor’ was counted as Manner as it clearly communicates the fact that the ball *roll* but did not *bounce* when travelling in space from the outside to the inside of the box. In the examples below, Manner tokens are underlined, Path tokens appear in CAPITALS, and the analysis of the elicitations is given in grey.

## Footnotes

1 See Thierry (2016) for experimental work that approaches the linguistic relativity question from a neurofunctional perspective.

2 F = Figure; G = Ground; Ma = Manner; Mo = Motion; P = Path.

3 Some researchers who use the term ‘linguistic relativity’ as a general label to refer to all possible ways in which language can affect non-verbal representations and processes maintain, naturally, that what I refer to as ‘language-as-strategy’ effects constitute evidence for linguistic relativity (cf. Lucy 2014: 28). If transient effects of language on cognition count as evidence for linguistic relativity, then relativity is an established fact which, it is worth noting, is not necessarily incompatible with the universalist thesis presented in “Introduction”. What is controversial is whether language can have detectable effects on non-verbal behaviour in situations in which language is neither being used overtly nor covertly (cf. Slobin 2003).

4 The exception to the rule is Filipović’s (2011) study, which did find an effect of language group on memory for Path and Manner. This study, however, did not use a verbal interference manipulation and, therefore, it is uncertain whether it can be taken to be evidence for relativity, something which Filipović herself acknowledges (2011: 482).

5 Serial presentation of the video clips, as in Gennari et al. (2002), was preferred over simultaneous presentation, as in Cardini (2010). The latter mode of stimulus presentation is, I believe, highly unnaturalistic, as participants are required to process two alternate clips (effectively, two ‘possible worlds’) at the same time and during an extremely short time frame. This raises concerns as to whether participants are even capable of accomplishing such a task accurately and whether the response pattern would stay the same if the experimental design allowed for a more focused examination of the stimuli.

6 Note that no motion-causing agent was visible in these experimental items.

7 Interrater reliability measures were taken to make sure that the coding of the verbal elicitations was accurate. Interrater reliability was calculated as percent agreement between two data collectors, as in McHugh (2012), on the basis of 25% of the dataset. The percent agreement score for *Manner info* was 92.65%, while the one for *Manner coded in main verb* was 90.12%.

8 Other studies have also found significant linguistic differences across language groups, yet weaker ones (cf. Gennari et al. 2002; Papafragou et al. 2008; Cardini 2010).

9 When I say ‘compelling evidence’, what I mean is evidence coming from a dual-task paradigm in which participants performed under conditions of verbal interference. Phillips & Boroditsky (2003), which looked at object conceptualisation in German vs. Spanish, is the only study that, to my knowledge, has found cross-group differences under conditions of verbal interference. However, the researchers themselves (2003: 932) call into question the results obtained: ‘[p]erhaps the shadowing task simply didn't disable all the aspects of language that could have been covertly recruited for the task? Perhaps some different, more complex verbal interference task would have changed the results’.
